# Targeting the PEDV 3CL protease for identification of small molecule inhibitors: an insight from virtual screening, ADMET prediction, molecular dynamics, free energy landscape, and binding energy calculations

**DOI:** 10.1186/s13036-023-00342-y

**Published:** 2023-04-18

**Authors:** Rajesh Kumar Pathak, Won-Il Kim, Jun-Mo Kim

**Affiliations:** 1 Department of Animal Science and Technology, Chung-Ang University, Anseong-si, Gyeonggi-do 17546 Republic of Korea; 2 College of Veterinary Medicine, Jeonbuk National University, Iksan, Jeollabuk-do 54596 Republic of Korea

**Keywords:** Pig, PEDV, Virtual screening, MD simulation, Veterinary drug

## Abstract

**Background:**

The porcine epidemic diarrhea virus (PEDV) represents a major health issue for piglets worldwide and does significant damage to the pork industry. Thus, new therapeutic approaches are urgently needed to manage PEDV infections. Due to the current lack of a reliable remedy, this present study aims to identify novel compounds that inhibit the 3CL protease of the virus involved in replication and pathogenesis.

**Results:**

To identify potent antiviral compounds against the 3CL protease, a virtual screening of natural compounds (n = 97,999) was conducted. The top 10 compounds were selected based on the lowest binding energy and the protein-ligand interaction analyzed. Further, the top five compounds that demonstrated a strong binding affinity were subjected to drug-likeness analysis using the ADMET prediction, which was followed by molecular dynamics simulations (500 ns), free energy landscape, and binding free energy calculations using the MM-PBSA method. Based on these parameters, four putative lead (ZINC38167083, ZINC09517223, ZINC04339983, and ZINC09517238) compounds were identified that represent potentially effective inhibitors of the 3CL protease.

**Conclusion:**

Therefore, these can be utilized for the development of novel antiviral drugs against PEDV. However, this requires further validation through in vitro and in vivo studies.

## Background

Porcine epidemic diarrhea (PED) is a swine disease caused by the emerging and re-emerging porcine epidemic diarrhea virus (PEDV); a virus, which belongs to the family Coronaviridae, genus Alphacoronavirus. The infected piglet will experience acute diarrhea, vomiting, and dehydration. The mortality rate of piglets on susceptible seronegative farms is almost 100% [1–3]. Even though older pigs have a lower mortality rate from PEDV infections, it can still impair growth [4]. The PED was initially identified in England and Belgium in 1971, and then in China in the early 1980s. However, since October 2010, a new highly pathogenic strain of PEDV has emerged in China and quickly spread into different parts of the world [1, 5, 6], causing significant financial losses to the farmers. There were approximately 8 million pig deaths in the United States caused by the epidemic between 2013–2014 [7].

Recent research suggests that, like SARS-CoV-2 and MERS-CoV, airborne transmission may also contribute to a PEDV outbreak [8, 9].The latter half of 2020 saw a regular occurrence of large-scale PEDV outbreaks on pig farms [6]. Yet, the protection provided by the commercially available inactivated and attenuated live PEDV vaccines is insufficient. Actually, vaccine immunity may not be adequate against epidemic strains despite elevated antibody titers following immunizations [6, 7]. Some picornaviruses and coronaviruses are effectively inhibited by GC376, a dipeptidyl bisulfite adduct salt. The previous study also demonstrated its effectiveness against PEDV 3CL protease [10]. As an antiviral medicine, it was tested in the cats infected by the viral disease Feline infectious peritonitis (FIP) caused by a feline coronavirus but was found to be associated with the side effect. It shows transient stinging at the injection sites, hair loss, subcutaneous fibrosis, and abnormal eruption of permanent teeth [11, 12]. Therefore, further studies are required before the use of GC376 as antiviral veterinary medicine. Alternative therapies to control this disease are urgently required, such as the discovery of antiviral drugs that are effective against PEDV.

The pathosystems of PEDV and pigs have been studied at the molecular level [9]. Ye et al. (2020) examined the interaction between 3CL protease and GC376. Using PyMOL and LigPlot, they identified several amino acid residues that were involved in the interaction. These included His41, Thr47, Phe139, Asn141, Gly142, Cys144, His162, Gln163, Leu164, Glu165, His171, Gln187, and Pro188. The researchers determined that these residues played a role in both catalysis and binding pocket formation, and were responsible for inhibiting the replication of PEDV [10]. The role of virus proteases in the replication and infection process has been well elucidated [13]. The development of viral protease inhibitors is an effective treatment for virus-borne infections such as HIV and HCV [14–16]. Therefore, the essential role of the 3CL protease in processing viral polyproteins for PEDV replication makes it an attractive antiviral drug target [17]. Natural compounds such as Tomatidine, Griffithsin, and Coumarin, alongside prenylated phenolic compounds, were reported to exhibit anti-PEDV activity in vitro. However, no anti-PEDV drugs are currently commercially available [9, 18–20].

**Fig. 1:**
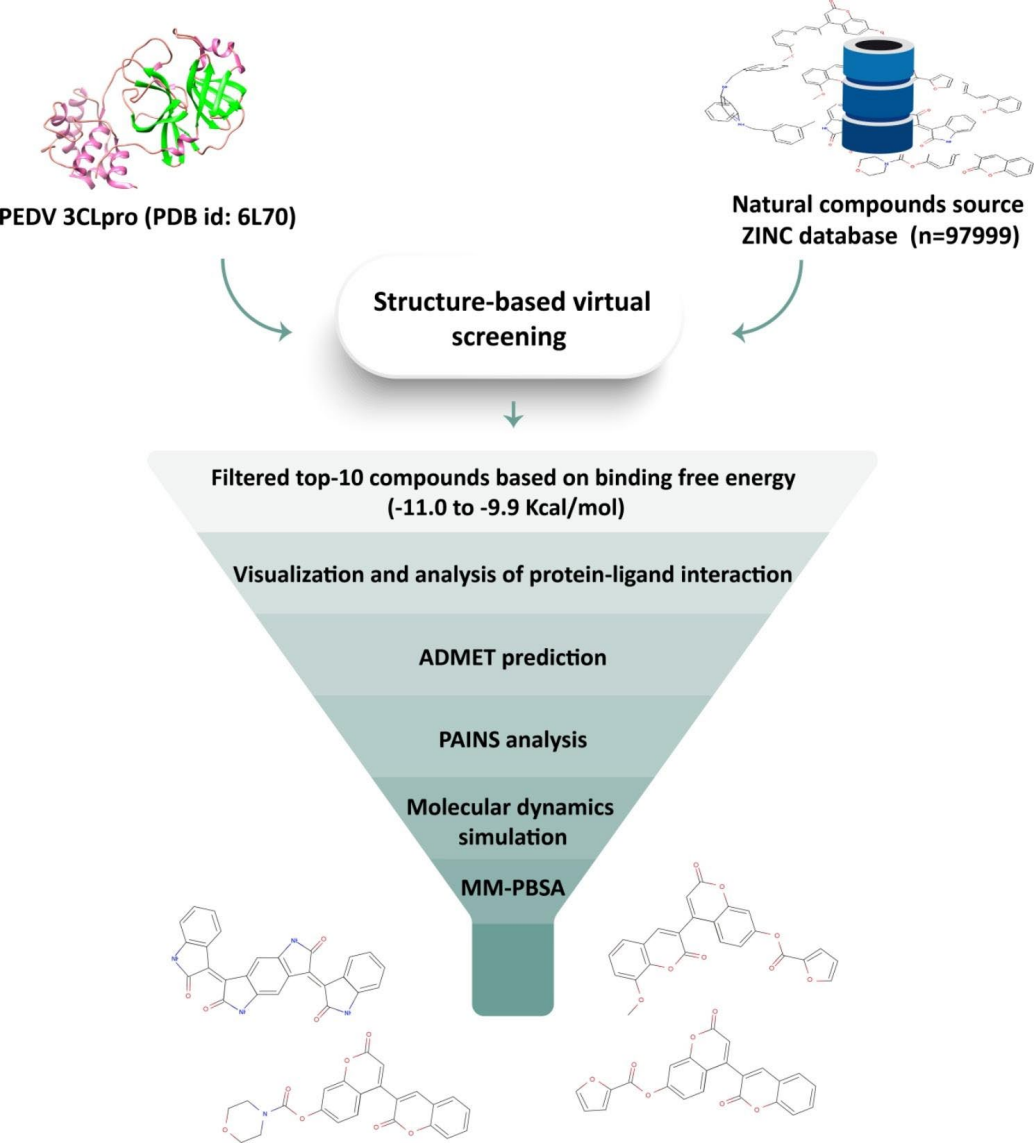
Summary of work conducted for identifying putative-lead compounds against 3CL protease of PEDV.

Previously, natural products were used to treat numerous complex diseases. The tremendous potential of natural products in antiviral activity is well documented in the literature [21, 22]. Thus, it was hypothesized that screening the natural product database against 3CL protease could provide the foundation to identify novel anti-PEDV drugs. Therefore, a computational high-throughput virtual screening approach was applied, which used a subset of natural compounds (n = 97,999) from the ZINC database against the PEDV 3CL protease to identify potential novel inhibitors. The top 10 compounds were sorted based on their binding energy with the 3CL protease and the protein-ligand interaction analyzed. Further, the top five compounds that demonstrated a strong binding affinity were employed for ADMET prediction, molecular dynamics simulation, Gibbs free energy landscape, and binding energy calculations through the MM-PBSA method. Consequently, novel small molecules were identified as 3CL protease inhibitors. Ultimately, providing a roadmap for the rapid discovery of future treatments against viral diseases in livestock animals (Fig. 1).

## Materials and methods

### Natural compounds datasets and target protein structure

A subset of natural compounds (n = 97,999) was downloaded from the ZINC database in the structure-data file format (SDF) [23], and OpenBabel (https://openbabel.org/wiki/Main_Page) was employed to convert all the compounds into PDBQT (Protein Data Bank (PDB), partial charge (Q), and atom type (T)) files. The co-crystallized structure of PEDV 3CL protease with a GC376 inhibitor (PDB ID: 6L70) was retrieved from the PDB database (https://www.rcsb.org). The protein structure was visualized and prepared with UCSF Chimera by removing all non-standard residues and co-crystallized ligand [24, 25]. Further, AutoDock tools were used to convert the pdbqt file of the target protein by adding charges and polar hydrogens. Subsequently, the grid box size was generated, which considered the binding cavity associated with the co-crystallized ligand [26]. To validate the docking parameters, we performed redocking of the co-crystalized ligand onto its receptor using AutoDock Vina. We then calculated the root-mean-square deviation (RMSD) value between the co-crystallized ligand (GC376) and its re-docked conformation using PyMOL [10, 26, 27].

### High-throughput virtual screening

The virtual screening method involves docking large numbers of compounds with their molecular targets of interest and uses drug discovery software to evaluate the binding free energy of the docked/screened compounds [28]. It is a computational technique widely used in the identification of lead molecules [29]. The molecular docking and virtual screening program AutoDock Vina identifies the preferred relative orientation of a ligand when docking or interacting with a molecular target and offers a protein-ligand complex structure with minimum binding energy [30]. Here, a shell script was used to perform a virtual screening of the natural compounds subset against the PEDV 3CL protease using AutoDock vina [30]. A python script was used to get the top 10 results and generated protein-ligand complexes using UCSF Chimera for visualization and analysis [25]. Further, protein-ligand interaction plots in 2D were produced using a Discovery Studio Visualizer (https://discover.3ds.com/discovery-studio-visualizer-download) to determine the amino acid residues involved in the interactions.

### ADMET and PAINS activity prediction

The drug-likeness properties (ADMET: absorption, distribution, metabolism, excretion and toxicity) of the top five screened compounds were analyzed. Information regarding molecular weight, LogP, H-bond donor and acceptor, and the topological surface area of each compound, alongside PAINS (Pan Assay Interference Compounds)-related information were obtained from the ZINC database [23]. Additionally, the toxic properties, which include, mutagenic, tumorigenic, and irritant were predicted by OSIRIS Property Explorer (https://www.organic-chemistry.org/prog/peo/).

### MD simulations

The top five compounds were chosen from the top 10 identified by virtual screening and drug-likeness analysis. The target protein 3CL protease was also obtained for molecular dynamics simulation. The MD simulation was conducted using GROMACS version 2018.1 [31, 32]. The system preparation was performed in accordance with previous research [33–35]. PRODRG was used to generate the ligand topology [36], whilst the pdb2gmx was used to create the protein topology [37]. Solvation was performed using the simple point charge water model. To construct the topologies for protein-ligand complexes, the topologies of proteins and ligands were merged. Cube-shaped boxes were generated, and complexes were placed inside of them. The electroneutrality of the system was preserved by using Na^+^ and Cl^−^ ions. Moreover, the energy minimization of protein and protein-ligand complexes was accomplished using the steepest descent minimization algorithm. The systems were also equilibrated using NPT and NVT simulations. Finally, all systems were subjected to a simulation time of 500 ns; coordinates were saved at 2 fs intervals. The trajectories of protein and protein-ligand complexes were examined for structural stability analysis by root-mean-square deviation (RMSD), Flexibility analysis by root-mean-square fluctuation (RMSF), compactness analysis by a radius of gyration (Rg), and solvent-accessible surface area analysis (SASA), Hydrogen bond analysis, in addition to, principal component analysis (PCA) was performed using GROMACS (https://www.gromacs.org/) utilities gmx ‘rms’, ‘rmsf’, ‘gyrate’, ‘sasa’, ‘hbond’, ‘covar’ and ‘anaeig’ respectively. The 2D plotting program Grace (https://plasma-gate.weizmann.ac.il/Grace/) was used to analyze and visualize the results.

### Free energy landscape (FEL) and binding energy calculation (MM-PBSA)

We determined the minima states of protein and protein-ligand complexes using the free energy landscape analysis (FEL). A GROMACS utility called ‘gmx sham’ was used to calculate the FEL. Further, a python script was used to visualize the results and produce 3D images [38]. High-throughput MD simulation data were used to execute binding free energy computations of selected protein-ligand complexes using the g_mmpbsa tool [39]. Free energy associated with the binding of the protein-ligand complex (G_binding_) can be represented as,

$$\varDelta {G}_{binding }= {G}_{complex }- ({G}_{protein}+{G}_{ligand})$$

Here, G_complex_ represents the total binding free energy of the complex, G_protein_ represents unbound receptor, and G_ligand_ represents unbound ligand, respectively. Furthermore, the contribution of amino acid residue ‘x’ energy involved in an interaction was calculated as:

$$\varDelta {R}_{x}^{BE}=\sum _{i=0}^{n}({A}_{i}^{bound}-{A}_{i}^{free})$$

Where, *n* denotes the total number of residues, and *A*_*i*_^*bound*^ and *A*_*i*_^*free*^ represent the energy of the i^th^ atom for each ‘x’ residue.

## Results

### Investigating putative hits for the development of PEDV 3CL protease inhibitors

It is possible to use virtual screening to discover the best intermolecular framework between macromolecular targets and small molecules, such as drugs via the screening of chemical compound databases. It predicts which ligand will interact optimally with a target to form a complex. The ligand is then sorted according to its binding free energy with the target. The present study demonstrated the utilization of ZINC database natural compounds (n = 97,999) to conduct a virtual screening against the PEDV 3CL protease to identify putative hits. The binding free energy of each compound included in the study was evaluated to identify putative hits for further evaluation. Generally, a protein-ligand complex with low binding energy has a high binding affinity. Therefore, the top 10 screened compounds exhibiting minimum binding energy with the 3CL protease were selected as putative lead compounds (-11.0 to -9.9 Kcal/mol). The binding energy of the co-crystallized ligand GC376 was − 7.5 kcal/mol, and the RMSD value between GC376 and its re-docked conformation was predicted to be 1.224 Å. This RMSD value serves as an indicator of the efficiency and validity of the docking protocol. The ZINC id, binding free energy, and interacting amino acid that contributed to the protein-ligand interactions of top-10 lead like compounds are listed in Table 1.

**Table 1:** List of the top 10 screened compounds, their binding free energies, and interacting amino acid residues of the PEDV 3CL protease. The amino acid residues in **bold** indicate the residues involved in conventional hydrogen bond interactions

SN	ZINC id	Binding energy (Kcal/mol)	Amino acid residues contributed to interactions
1.	ZINC38167083	-11.0	**Arg130**, Leu198, Tyr280, Ser282, **Leu283**, Asp285
2.	ZINC09517223	-10.4	His41, Thr47, **Asn141**, Leu164
3.	ZINC03960761	-10.3	His41, Thr47, Gln163, Leu164, Glu165, **Gln187**, Thr189
4.	ZINC04339983	-10.2	His41, Thr47, Ile140, **Asn141**, Ala143, Cys144, Leu164, Glu165
5.	ZINC09517238	-10.1	His41, Thr47, Ile140, **Asn141**, Ala143, Cys144, Leu164, Glu165, Leu190
6.	ZINC05220992	-10.0	Gly23, Asn24, Met25, **Ile43**, Ala44, Ser45, Ile51, Asp52, Ala56, Val59
7.	ZINC11866257	-10.0	His41, Ile140, **Asn141**, Ala143, Cys144, Leu164, Glu165, Leu190
8.	ZINC15958854	-10.0	His41, Ile140, Ala143, Cys144, **His162**, **Gln187**, Pro188, Leu190
9.	ZINC03846598	-9.9	**His41, Thr47, ILE140, Asn141, Gly142**, Cys144, Leu164, Glu165
10.	ZINC08918302	-9.9	**Arg136**, Leu198, Asp285

### Evaluation and visualization for the binding of lead compounds with 3CL protease

**Fig. 2:**
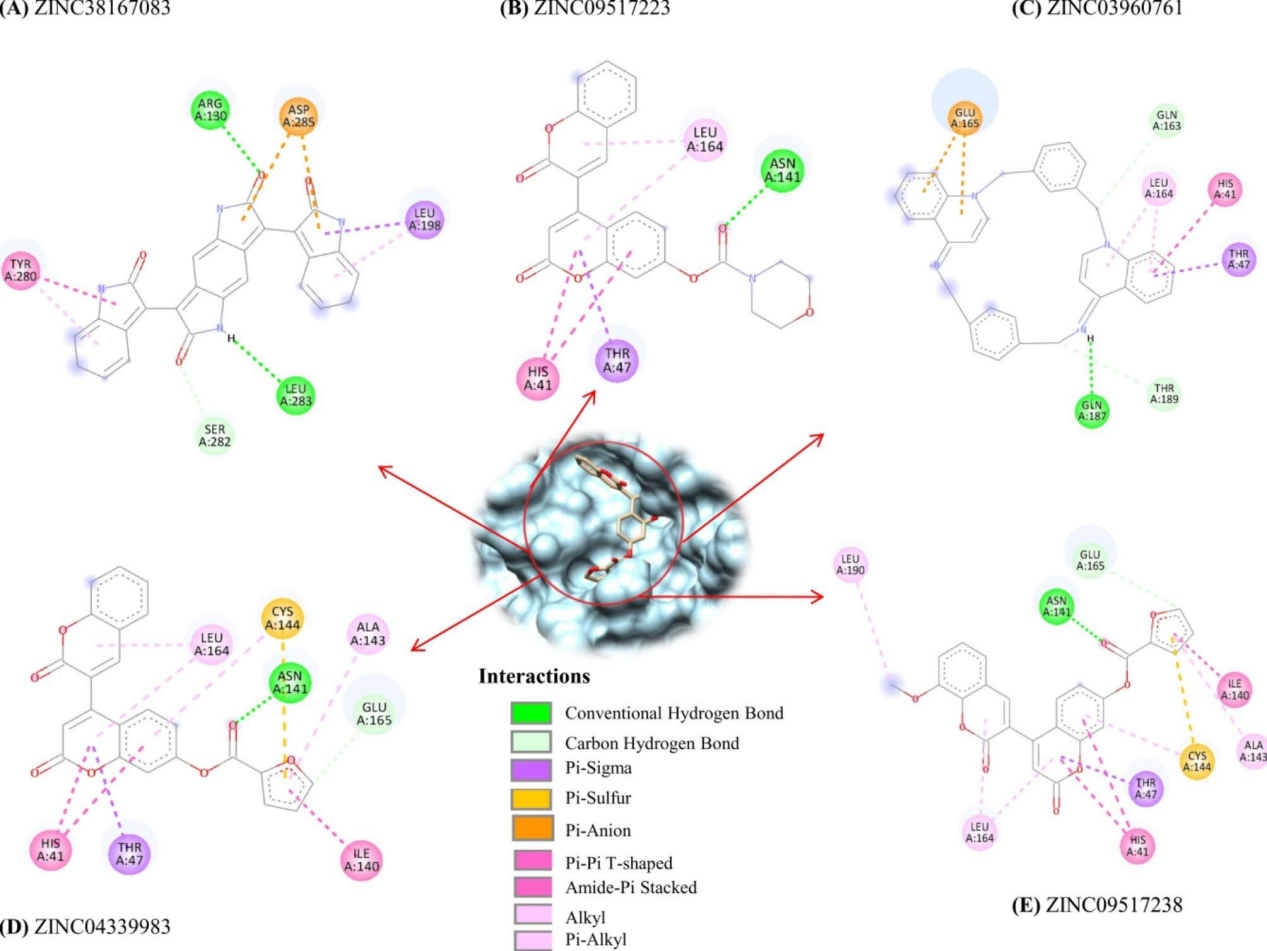
2D representation of the binding interactions of the top five screened natural compounds with PEDV 3CL protease depicted key amino acid residues that contributed to protein-ligand interactions. (**A**) ZINC38167083, (**B**) ZINC09517223, (**C**) ZINC03960761, (**D**) ZINC04339983, (**E**) ZINC09517238

Virtual screening offers the best interacting compounds with macromolecular targets based on binding free energy. Of the top 10 screened compounds, the top five were selected (binding energy range: -11.0 to -10.1 kcal/mol) for further analysis using different parameters to evaluate its binding nature and inhibitory potential with the 3CL protease. ZINC38167083 was found to interact with Arg130 and Leu283 with a conventional hydrogen bond; forming one pi-sigma and one alkyl bond with Leu198; one pi-pi t-shaped and one pi-alkyl with Tyr280, one carbon-hydrogen bond with Ser282, and two pi-anion bonds with Asp285 with 3CL protease and a binding free energy of -11.0Kcal/mol (Fig. 2A). Moreover, ZINC09517223 interacted with the 3CL protease and Asn141, which has one conventional hydrogen bond; His41 with two pi-pi t-shaped bonds; Thr47 with one pi-sigma bond and Leu164 with two pi-alkyl bonds and a binding free energy of -10.4 Kcal/mol (Fig. 2B). ZINC03960761 interacted with 3CL protease with a binding energy of -10.3 Kcal/mol and formed one conventional hydrogen bond with Gln187; one carbon-hydrogen bond with Gln163 and Thr189; one pi-pi t-shaped bond with His41 and one pi-sigma bond with Thr47, as well as two pi-alkyl bonds with Leu164 (Fig. 2C). ZINC04339983 interacted with 3CL protease with a binding energy of -10.2 Kcal/mol and formed one conventional hydrogen bond with Asn141, and one carbon-hydrogen bond with Glu165; two pi-pi t-shaped bonds with His41; one pi-sigma bond with Thr47; one amide-pi stacked bond with Ile140; one pi-alkyl bond with Ala143; one pi-alkyl and pi-sulfur bonds with Cys144, as well as two pi-alkyl bonds with Leu164 (Fig. 2D). ZINC09517238 interacted with the 3CL protease with a binding energy of -10.1 Kcal/mol. It formed one conventional and one carbon-hydrogen bond with Asn141 and Glu165, respectively. It also interacted with His41 with two pi-pi t-shaped bonds; Thr47 with one pi-sigma bond; Ile140 with one amide-pi stacked bond; Ala143 with one pi-alkyl bond; Cys144 with one pi-alkyl and one pi-sulfur bond; Leu164 with two pi-alkyl bonds; Besides, it formed one alkyl bond with Leu190 (Fig. 2E). Whereas, the interaction of reference compound i.e. co-crystallized ligand GC376 was also analyzed. It was interacted with 3CL protease Phe139, His162, and Glu165 with conventional hydrogen bonds; His41 with alkyl bond; Leu164 with alky and pi-alkyl bonds as well as Leu166 with pi-alkyl bond but binding energy was observed as higher (-7.5 Kcal/mol) as compared to identified natural putative lead compounds.

### Assessment of drug-likeness through the physicochemical property, toxicity, and PAINS analysis

The drug-likenesses of the top five selected compounds were analyzed based on ADMET analysis followed by PAINS. In any drug discovery program, ADMET analysis represents a fundamental task to investigate the chemical nature of the compounds. Therefore, different parameters such as molecular weight, LogP, H-bond donor and acceptor, topological polar surface area, mutagenic, tumorigenic, and irritant properties as well as the PAINS of each selected compound were analyzed. ADME and PAINS-related information were extracted from the ZINC database. In addition, the OSIRIS Property Explorer tool was used to predict toxicity (T). Based on our analysis, the top-screened selected compounds exhibited drug-like properties but PAINS-related problem in ZINC03960761 and irritation in ZINC09517238 was detected during PAINS and toxicity analysis, respectively. Besides, all the selected compounds also possessed polar surface areas < 140 Å^2^, indicating high cell membrane permeability. The results obtained during the drug-like analysis are depicted in Table 2.

**Table 2:** Physicochemical properties and drug-likeness from the identified compounds

S.N.	Descriptors	ZINC38167083	ZINC09517223	ZINC03960761	ZINC04339983	ZINC09517238
1.	Molecular Weight (g/mol)	446.422	419.389	494.642	400.342	430.368
2.	LogP	3.317	3.397	6.202	4.379	4.387
3.	H-bond donor	4	0	2	0	0
4.	H-bond acceptor	8	7	2	7	8
5.	Topological Polar Surface Area (Å²)	131	99	31	99	109
6.	Mutagenic	No	No	No	No	No
7.	Tumorigenic	No	No	No	No	No
8.	Irritant	No	No	No	No	Yes

### Structural and conformational analysis of the 3CL protease in unbound and bound systems during MD simulation

To visualize the dynamic behavior of the 3CL protease both before (unbound) and after ligand binding (bound), a 500 ns MD simulation was conducted. Various parameters i.e., RMSD, RMSF, Rg, SASA, H-bond, PCA, FEL, and the binding free energy calculation were used to summarize the results.

#### Stability analysis

**Fig. 3:**
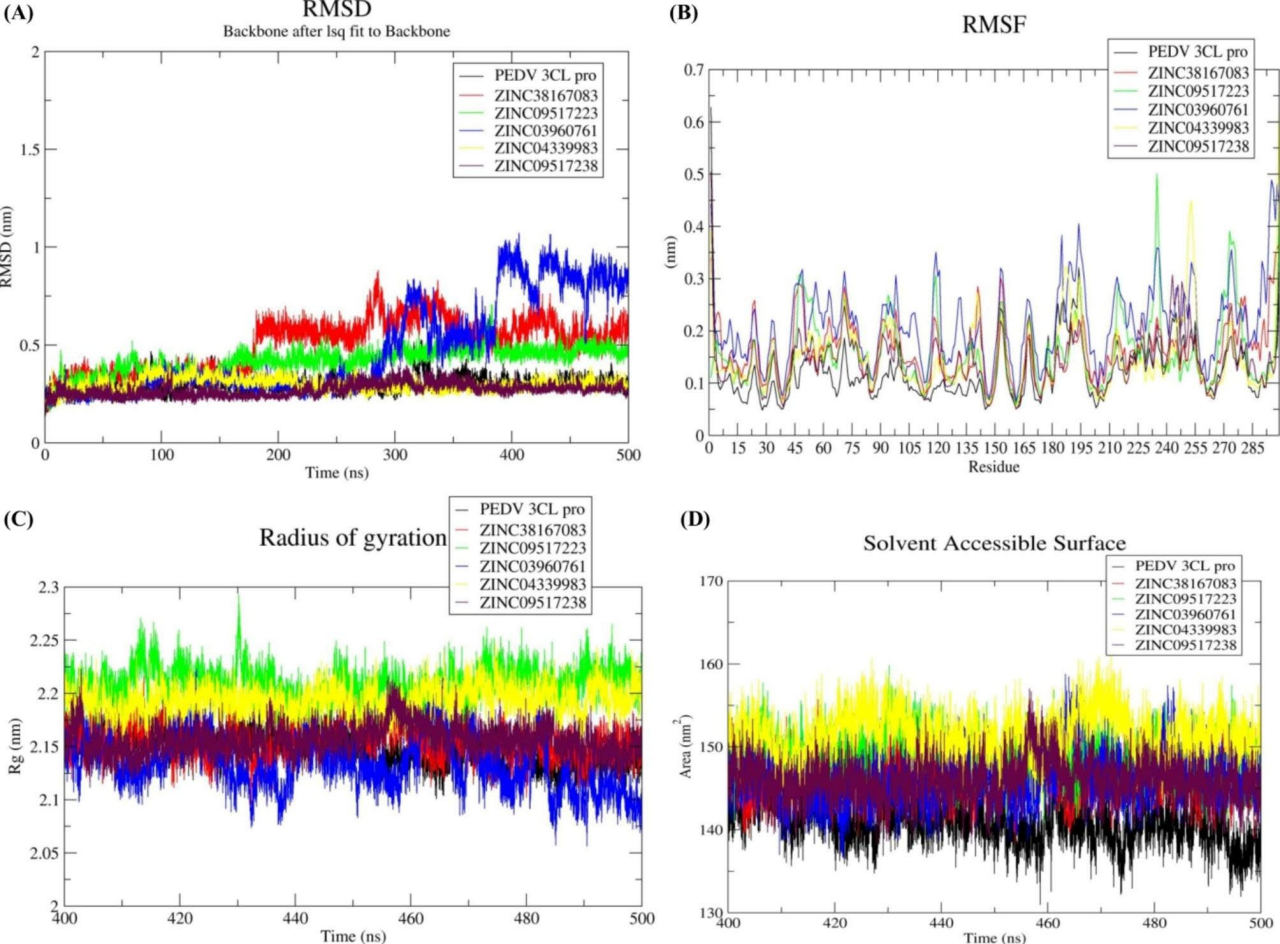
Stability analysis (**A**) RMSD values for the PEDV 3CL protease-compound complexes. Flexibility analysis **(B)** RMSF values for the PEDV 3CL protease-compound complexes over the final 100 ns of the simulations. Compactness (**C**) Rg, and Solvent accessible surface area analysis (**D**) SASA values for the final 100 ns of the simulations. Black, red, green, blue, yellow, and maroon colors represent the PEDV 3CL proteases, ZINC38167083, ZINC09517223, ZINC03960761, ZINC04339983, and ZINC09517238, respectively

RMSD was used to measure conformational stability during the MD simulation of proteins in relation to their structure. Specifically, structures with smaller RMSD values are more stable than those with larger RMSD values. We have computed the RMSD value for 500 ns (Fig. 3A). Deviation from initial to next and subsequent structures is represented by it. Here, based on the RMSD graph of backbone c-alpha atoms, the 3CL protease, and all the complexes exhibited fewer fluctuations with low RMSD values following 400 ns. The average RMSD of the 3CL protease was calculated as 0.26 nm. Moreover, the RMSD values of the 3CL protease-ZINC38167083, 3CL protease-ZINC09517223, 3CL protease-ZINC03960761, 3CL protease-ZINC04339983, and 3CL protease-ZINC09517238 were 0.49, 0.41, 0.47, 0.29, and 0.26 nm, respectively. It was predicted that all complexes got established after 400 ns. Hence, the final 100 ns trajectories were considered for further analysis.

#### Flexibility analysis

Proteins maintain their properties by being flexible, which can be accessed through RMSF analysis. The RMSF of the 3CL protease and their complexes were therefore analyzed for the last 100 ns equilibrated trajectory. This indicates amino acid residue fluctuations upon binding of ligands, here higher fluctuations were observed for amino acid residues from positions 180 to 299 than for amino acid residues from positions 1 to 179. Although the mean RMSF value for most amino acid residues was below 0.4 nm. Besides, Ala1, Gly298, and Val299 exhibited higher RMSF mean values (Fig. 3B). The average RMSF value of the 3CL protease was measured as 0.12 nm. Moreover, the RMSF values of the 3CL protease-ZINC38167083, 3CL protease-ZINC09517223, 3CL protease-ZINC03960761, 3CL protease-ZINC04339983, and 3CL protease-ZINC09517238 were 0.16, 0.15, 0.21, 0.14 and 0.13 nm, respectively (Fig. 3B). Overall, lower fluctuation was observed except for the 3CL protease-ZINC03960761 complex, supporting their utilization as the putative lead compounds.

#### Compactness analysis

To understand protein structure compactness, stability, and folding, the Rg values can be calculated over time. The 3CL protease and their complexes were analyzed for Rg values to determine their structural compactness. The Rg values of the 3CL proteases were calculated and plotted, the 3CL protease-ZINC38167083, 3CL protease-ZINC09517223, 3CL protease-ZINC03960761, 3CL protease-ZINC04339983 and 3CL protease-ZINC09517238 systems from the final 100 ns MD trajectories. The average Rg values were calculated as 2.14, 2.15, 2.20, 2.13, 2.19, and 2.15 nm, respectively. The Rg results indicate that the 3CL protease-ZINC03960761 exhibits a more compact structure than the other complexes (Fig. 3C).

#### Solvent accessible surface area (SASA) analysis

Ligand-induced solvent-accessible area changes were determined using a SASA analysis from the final 100 ns of the simulation (Fig. 3D). The average SASA value for the 3CL proteases, 3CL protease-ZINC38167083, 3CL protease- ZINC09517223, 3CL protease–ZINC03960761, 3CL protease-ZINC04339983, and 3CL protease-ZINC09517238 was calculated as 140, 146, 148, 146, 151, and 146 nm^2^, respectively. Here, the SASA value of the 3CL protease-ZINC04339983 complex was higher than the others. Besides, a similar type of pattern was observed for all the systems (Fig. 3D), revealing comparatively least changes following the binding of each compound.

#### Hydrogen bonds (HBs) analysis

**Fig. 4:**
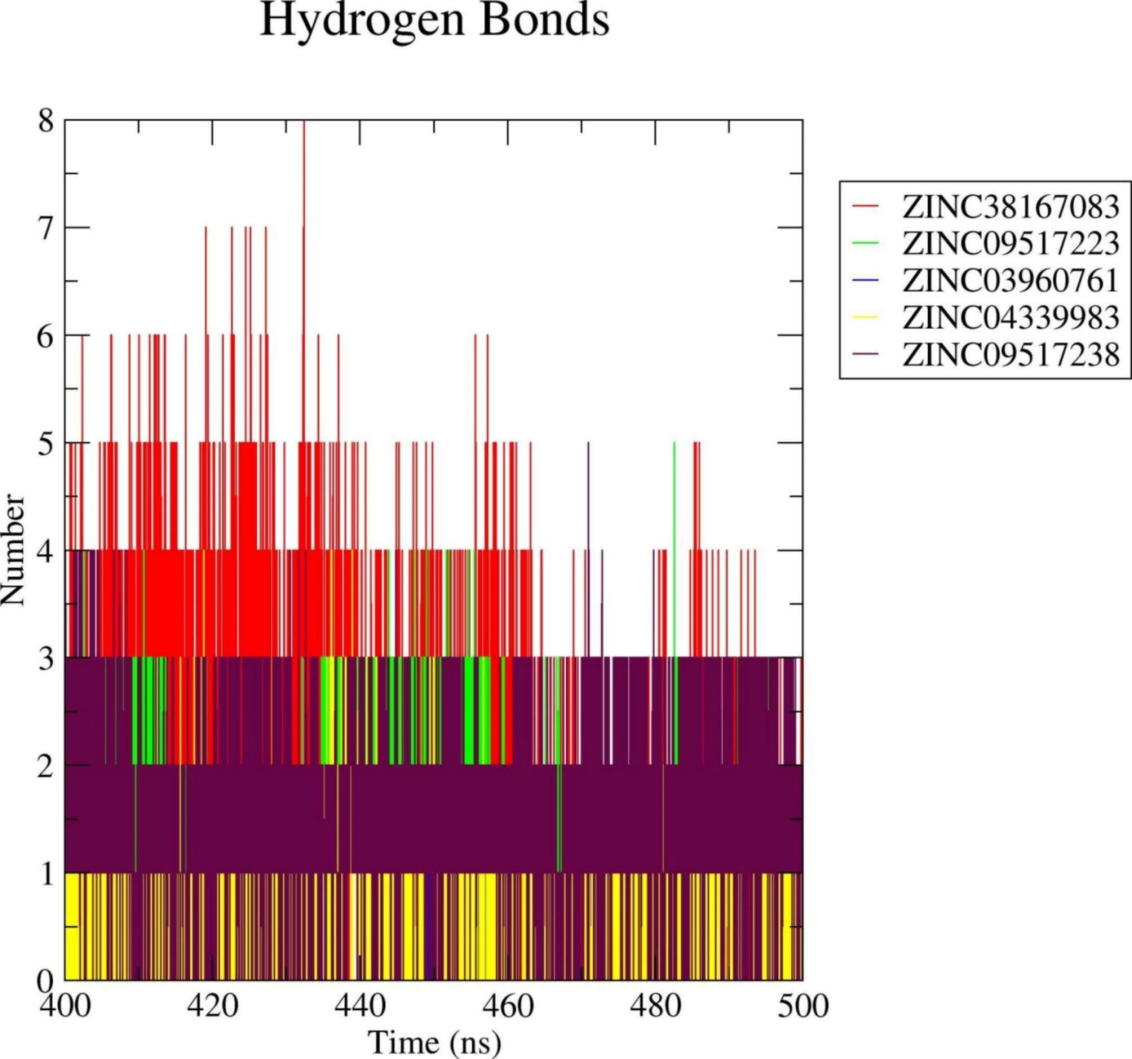
Changes in the number of hydrogen bonds in each respective complex according to data from the final 100 ns of the simulations. Red, green, blue, yellow, and maroon colors represent ZINC38167083, ZINC09517223, ZINC03960761, ZINC04339983, and ZINC09517238, respectively

The most crucial bond that stabilizes the protein-ligand interactions is the hydrogen bond. Therefore, we counted the number of hydrogen bonds produced from the natural compounds’ interaction with the target 3CL protease. Here, the 3CL protease-ZINC38167083 exhibited the highest number (0–8) of hydrogen bonds compared to the estimated complexes (Fig. 4).Comparatively, the 3CL protease-ZINC09517223 complex showed an average of 0–5 hydrogen bonds; the 3CL protease-ZINC03960761 complex averaged 0–2 hydrogen bonds; 3CL protease-ZINC04339983 averaged 0–4 hydrogen bonds, and the 3CL protease-ZINC09517238 averaged 0–5 hydrogen bonds during the final 100 ns (Fig. 4). Thus, during the protein-ligand interactions, these compounds interacted with the 3CL protease to produce a stable complex.

#### Principal component analysis (PCA)

**Fig. 5:**
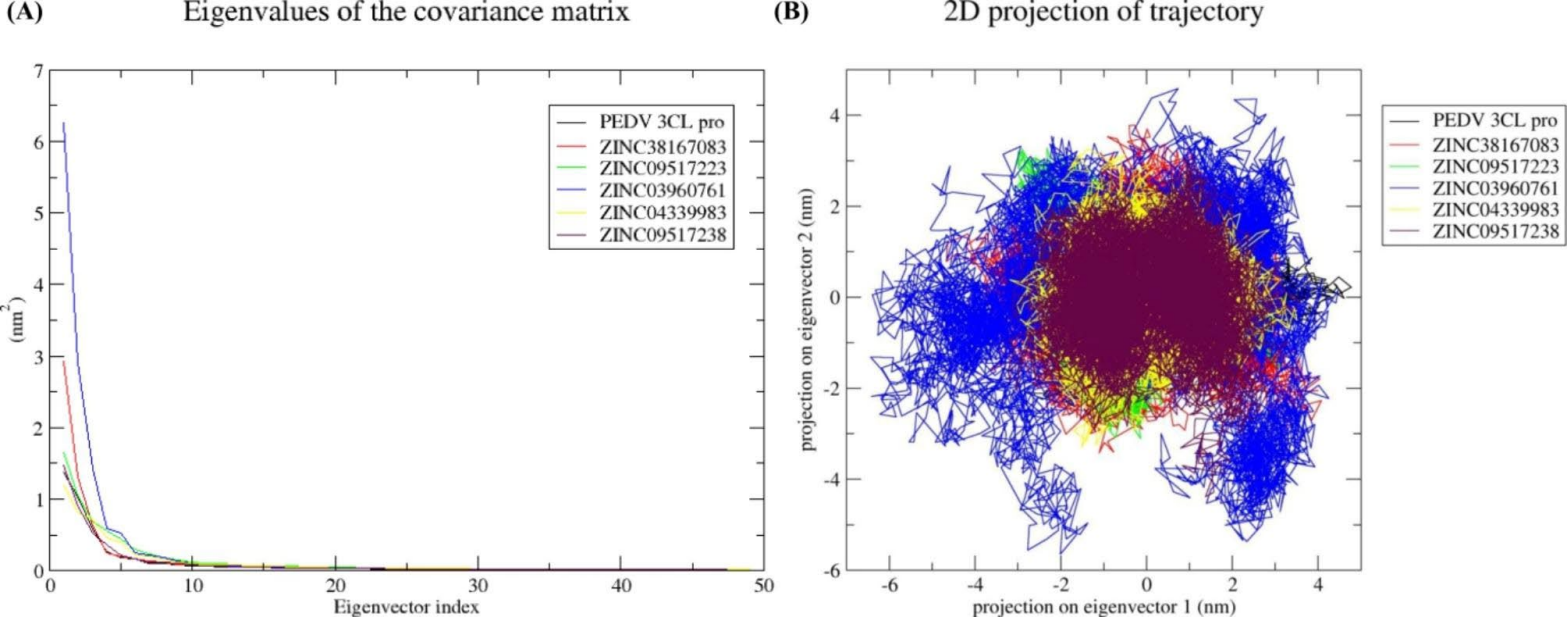
Principal component analysis. **(A)** Eigenvalues derived from the final 100 ns of each simulation and used for PCA depicted eigenvalues vs. the first fifty eigenvectors. (**B)** First two eigenvectors depicted the PEDV 3CL protease motion in space for all the systems. Black, red, green, blue, yellow, and maroon colors represent PEDV 3CL protease, ZINC38167083, ZINC09517223, ZINC03960761, ZINC04339983, and ZINC09517238, respectively

PCA analyses were conducted to capture significant conformational changes during ligand binding. A protein’s overall motion is determined primarily by the first few eigenvectors. Therefore, changes in the structural movement were analyzed using the first 50 eigenvectors (Fig. 5A). The percentage-wise correlated motions were calculated from the initial 10 eigenvectors and provided a clear understanding of the motions induced by the ligand binding. The 3CL proteases, 3CL protease-ZINC38167083, 3CL protease- ZINC09517223, 3CL protease-ZINC03960761, 3CL protease-ZINC04339983 and 3CL protease-ZINC09517238 showed 69.57%, 76.18%, 64.51%, 84.11%, 62.45% and 68.17% correlated motions, respectively. Here, we can see that the 3CL protease-ZINC09517223 and 3CL protease-ZINC04339983 complexes showed the lowest motions.

Figure 5 illustrates that the first few eigenvectors reflect the overall dynamics of the protein. This led to the selection of the first two eigenvectors and their plotting in phase space (Fig. 5B). The 3CL protease, 3CL protease-ZINC38167083, 3CL protease- ZINC09517223, 3CL protease-ZINC04339983, and 3CL protease-ZINC09517238 clusters are the most stable (low correlated motions) when compared to the 3CL protease–ZINC03960761(Fig. 5B).

### Gibbs free energy landscape (FEL) analysis

**Fig. 6:**
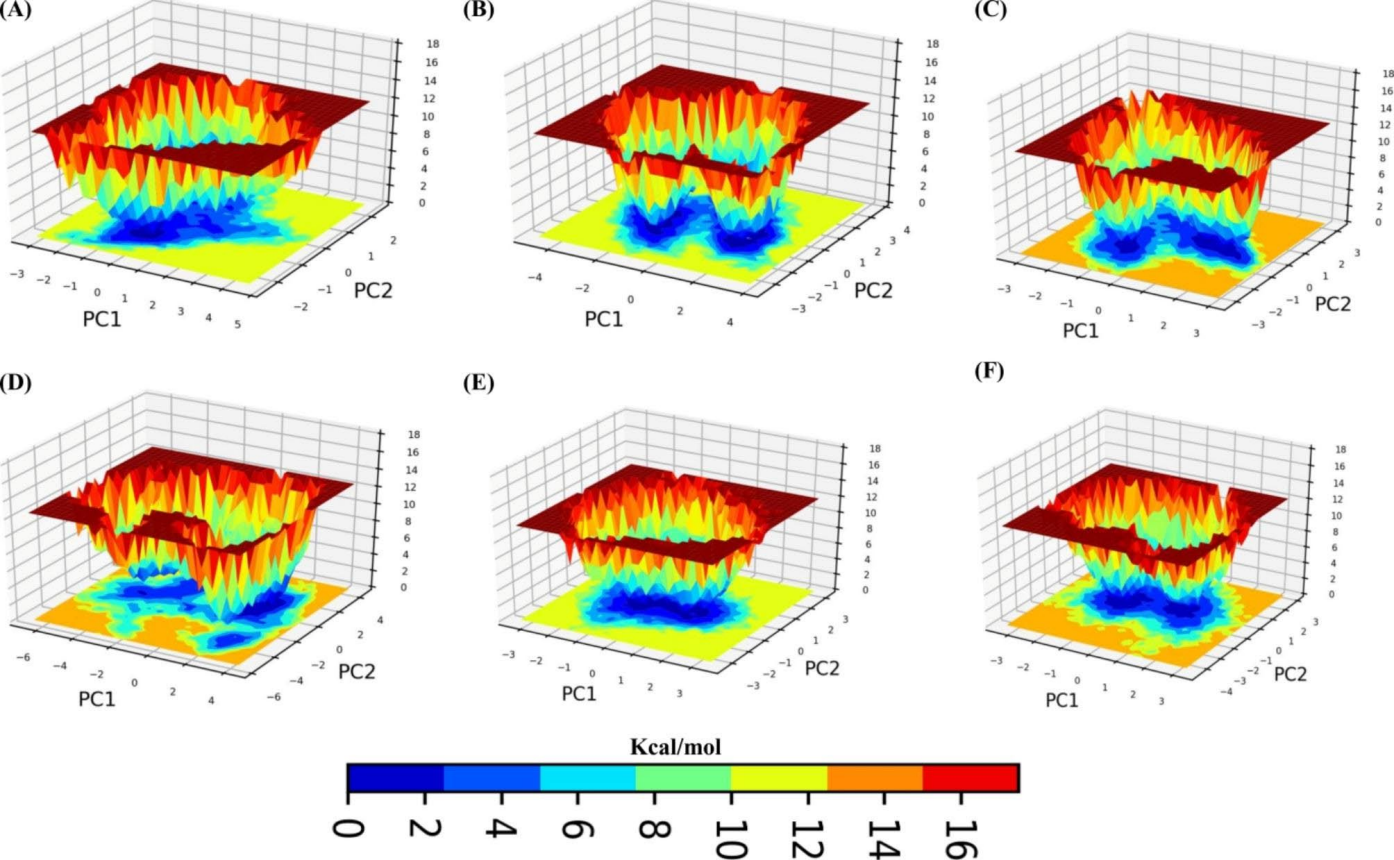
The color-coded illustration of the Gibbs free energy landscape (FEL) was plotted using PC1 and PC2. The color bar indicates the Gibbs free energies (kcal/mol) for conformational states with the lowest (blue) and highest (red) energies. (**A**) PEDV 3CL protease (**B**) 3CL protease-ZINC38167083, (**C**) 3CL protease-ZINC09517223, (**D**) 3CL protease-ZINC03960761, (**E**) 3CL protease-ZINC04339983, and (**F**) 3CL protease-ZINC09517238 docked complexes

Gibbs free energy landscape calculations were performed using the first two principal components. Figure 6 illustrates the FEL calculated for all the systems. The color bar represents the Gibbs free energies (kcal/mol) ranging from the lowest energy (blue) to the highest energy (dark yellow) conformational states. In the case of 3CL proteases, 3CL protease-ZINC38167083, 3CL protease-ZINC09517223, 3CL protease-ZINC04339983, and 3CL protease-ZINC09517238, the enriched energy minima with wide space have been observed (blue color). These cover a larger blue area as compared to the 3CL protease–ZINC03960761 complex and represent a stable cluster (Fig. 6). Moreover, there are several minima in the conformational space of 3CL protease-ZINC03960761 (Fig. 6D). Additionally, FEL analysis demonstrated that all the complexes gained minimum energy corresponding to the most stable conformations.

### Binding free energy calculation

An MM-PBSA method was used to estimate the binding free energy of all simulated complexes to validate their binding affinities. Binding free energies were calculated using the last 5 ns of the MD simulation trajectories. The calculated binding free energy for the 3CL protease-ZINC38167083, 3CL protease-ZINC09517223, 3CL protease-ZINC03960761, 3CL protease-ZINC04339983, and 3CL protease-ZINC09517238 complexes were − 104.208, -133.292, -176.620, -168.155 and − 153.980 kJ mol^− 1^, respectively. The calculated values for the van der Waals, electrostatic, polar solvation, SASA, and binding free energies are given in Table 3.

**Fig. 7:**
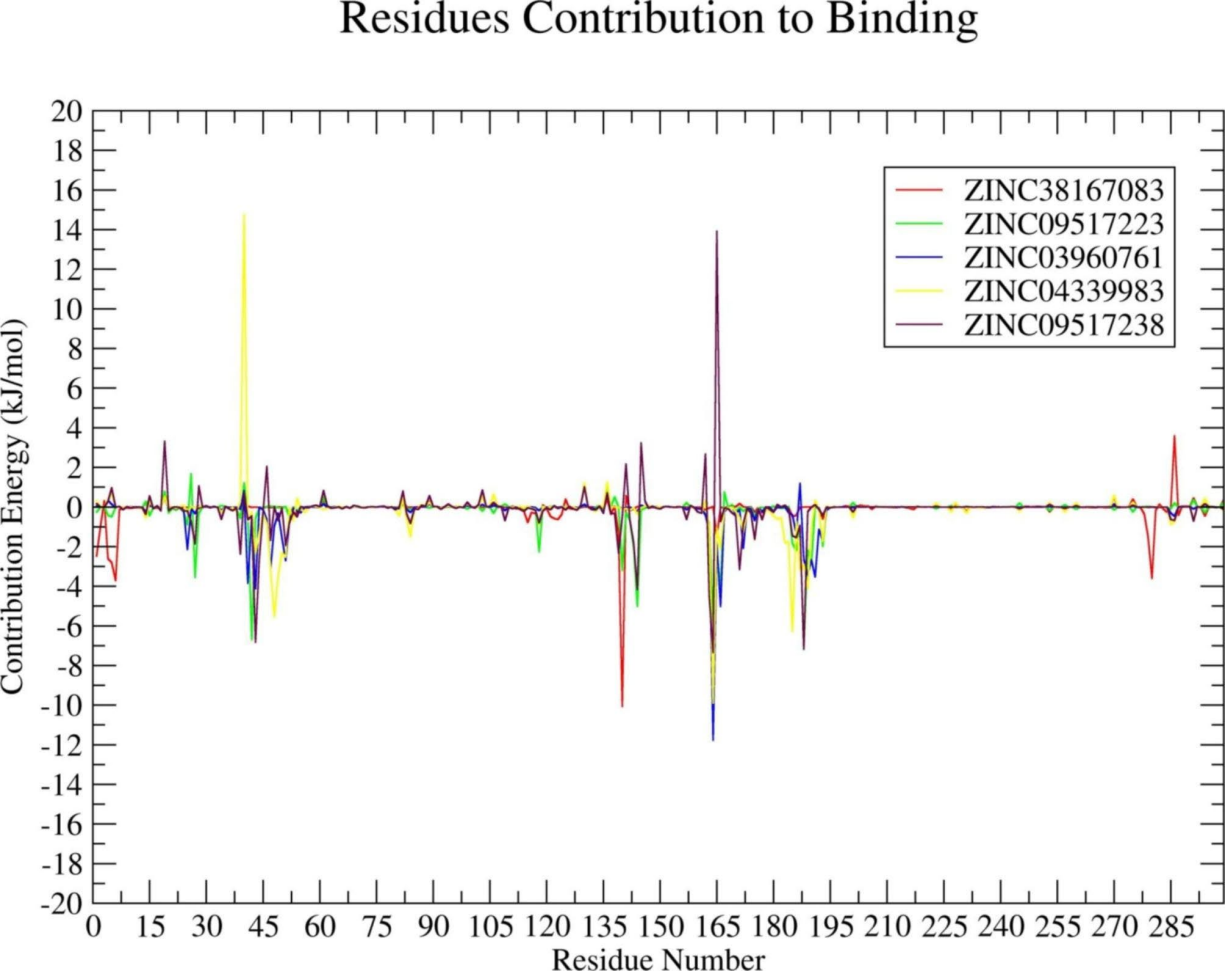
Plot depicting the amino acid residues in the PEDV 3CL proteases contributing to the binding with natural compounds. Red, green, blue, yellow, and maroon colors represent ZINC38167083, ZINC09517223, ZINC03960761, ZINC04339983, and ZINC09517238, respectively

Residual binding energy analyses were conducted for the simulated complexes to identify the amino acid residues that are crucial for ligand binding. All the selected compounds were observed to be significantly involved in the protein-ligand interactions with amino acid residues of the 3CL protease, which indicates a potential for 3CL protease inhibitors. The amino acid residues from positions 1 to 50 and 135 to 195 contributed more to interactions (Fig. 7).

**Table 3:** Affinities of natural compounds with a PEDV 3CL pro (van der Waals and electrostatic forces, polar solvation, SASA, and binding free energy in kJ mol^-1^)

Compound name	van der Waals energy	Electrostatic energy	Polar solvation energy	SASA energy	Binding energy
ZINC38167083	-145.387 ± 16.665	-64.348 ± 14.516	120.788 ± 23.081	-15.260 ± 1.329	-104.208 ± 19.031
ZINC09517223	-160.024 ± 10.439	-17.024 ± 6.347	59.090 ± 8.067	-15.334 ± 0.969	-133.292 ± 9.667
ZINC03960761	-208.091 ± 15.088	0.256 ± 0.838	49.904 ± 7.742	-18.689 ± 1.384	-176.620 ± 16.265
ZINC04339983	-253.374 ± 11.038	-23.315 ± 9.467	130.274 ± 15.800	-21.740 ± 0.901	-168.155 ± 13.922
ZINC09517238	-252.970 ± 13.270	-36.563 ± 11.736	156.404 ± 19.576	-20.851 ± 0.883	-153.980 ± 12.737

## Discussion

The global swine industry faces a heavy economic burden due to outbreaks of PEDV [6]. There are currently no commercial vaccines that are effective against PEDV variants due to their high virulence nature. Additionally, there are no antiviral drugs available to treat this disease [6, 40]. This necessitates the development of effective and safe strategies to combat the virus. The use of natural compounds for the treatment of complex diseases dates back to ancient times, and there are many therapeutic ingredients found in nature. Due to advancements in scientific technology, our knowledge and use of natural compounds for therapeutic purposes are continually improving [21]. Previous research has shown that the PEDV infection in Vero cells was reduced by Griffithsin, a mannose-specific lectin that prevents viral attachment and disrupts cell-to-cell transmission [18]. Besides, in vitro antiviral effects of phenol-prenylated compounds from the leaves of *Sabia limoniacea* on PEDV replication have been demonstrated, suggesting the antiviral potential of the natural products against PEDV [20]. Therefore, the natural compounds database can be utilized to screen against viruses for investigating novel antiviral compounds. It is now established that viral proteases play a vital role in viral lifecycles, which proves it can be an ideal drug target for the investigation of antiviral compounds [10]. Drugs targeting individual viral proteases have been developed that are remarkably potent to combat significant human pathogens such as HIV and HCV [10]. Therefore, the PEDV 3CL protease is recommended as a possible molecular target for drug discovery [9, 10]. Virtual screening, ADMET, MD simulation, FEL, and binding energy calculations were used in the present study to identify possible lead compounds against PEDV. Virtual screening, a method of structure-based drug discovery investigates important lead molecules from a large compound database based on the lowest binding energy required to stabilize protein-ligand interactions [41].

From the structure-based virtual screening, the top ten natural compounds that interact with key amino acid residues of 3CL protease were selected. After visualizing and analyzing protein-ligand interactions, the top five compounds were selected and subjected to ADMET prediction, since it serves as an essential requirement for testing any candidate molecule [42]. As per Lipinski’s rule, a candidate drug molecule should have a molecular weight of fewer than 500 Daltons, a logP value < 5, hydrogen bond donors < 5, and hydrogen bond acceptors < 10 [43]. Our predicted lead compounds all passed these Lipinski filters; while, the topological surface area of the top 5 compounds was calculated, and found within an appropriate range i.e.,<140 Å^2^, which is indicative of high cell membrane permeability. Moreover, the toxicity prediction and PAINS analysis demonstrated the drug-confirmed behavior of the top 5 selected compounds. Besides, a PAINS-related problem was detected in ZINC03960761, and ZINC09517238 that passes all the criteria but an irritation-related problem was predicted during toxicity analysis. PAINS more commonly affects a number of biological targets non-specifically rather than specifically affecting one desired target [44]. Therefore, ZINC03960761 may cause toxicity.

To assess the overall stability of each predicted lead compound, MD simulations were conducted. It is a powerful method of predicting how macromolecules will behave before and after binding to ligands [34]. In addition, the simulated data were used to calculate the binding free energy of each compound over time, followed by the contribution of the amino acid residues of the 3CL protease to stabilize the protein-ligand complex. RMSD analysis showed that all systems were stabilized after 400 ns, suggesting a significant interaction between the predicted lead compounds and the 3CL protease. Therefore, the final 100 ns trajectories were utilized to analyze other parameters such as RMSF, Rg, SASA, H-bonds, PCA, and FEL to determine the nature of each compound. Based on the analysis, it was concluded that the ligand binding changes both the protein conformation and the dynamics required for the inhibition.

Further analysis of the natural compounds’ binding affinity towards the 3CL protease was conducted using the MM-PBSA binding free energy and residual binding energy calculations. This method represents a commonly used and well-accepted approach for calculating the binding free energy of protein-ligand complex data obtained from MD simulation results [39, 45]. By measuring the ligand binding affinity, which is directly related to the ligand potency, the strength of the binding contact between each compound and the 3CL protease was determined. Its evaluation is important in the field of drug discovery. Additionally, the free energy in the favorable reactions is negative. Therefore, lowering the binding energy enhances the interactions, while the high binding affinity of the protein-ligand complexes is correlated with the lower binding free energy [46]. Based on MM-PBSA and residual binding energy calculations, it was concluded that all the analyzed complexes i.e., 3CL protease-ZINC38167083, 3CL protease-ZINC09517223, 3CL protease-ZINC03960761, 3CL protease-ZINC04339983, and 3CL protease-ZINC09517238 were energetically stable. Previous studies confirmed that the 3CL protease amino acid residues Cys144, His162, Gln163, and Glu165 interacted with GC376 via hydrogen bonding. However, His41, Thr47, Phe139, Asn141, Gly142, Leu164, His171, Gln187, and Pro188 were involved in hydrophobic contacts [10]. These amino acid residues were reported as catalytic and binding pocket residues and were found to be responsible for the inhibition of PEDV replication. [10]. Figure 7 shows that amino acid residues at positions 01 to 50 and 135 to 195 contributed significantly to the binding with identified natural compounds, and all catalytic and binding pocket residues fall in this range. Therefore, it could act as a potential lead for the development of antiviral therapeutics against PEDV [47].

Advances in structural biology, bioinformatics, and vetinformatics are facilitating researchers to discover putative veterinary drugs by screening natural compound databases against macromolecular drug targets [35, 48]. This will save experimental cost and time, as well as boost research productivity [28]. Since the majority of medicines on the market come from natural sources or are derived from chemicals that are found in nature [21, 49]. Moreover, the ability of natural compounds to combat pathogenic viruses is well documented in the literature [49–51]. Therefore, the findings in the present investigation are informative in developing antiviral drugs for the treatment of pigs against PEDV infection.

## Conclusion

Finding new and potent antiviral drugs is necessary since PEDV infection is a major threat to the world’s swine industry. A number of computational methods were employed in the present study to find effective inhibitors that can target the 3CL protease of the virus. This computational study indicates that ZINC38167083, ZINC09517223, ZINC04339983, and ZINC09517238 have the potential to be developed as antiviral drugs against PEDV. Contrastingly, while the ZINC03960761 compound was similarly found to work as an interactome with the 3CL protease, it was not recommended as the lead due to the detection of associated PAINS-related problems. In the future, the activity of the recommended compounds can be optimized to enhance the potential of their antiviral activity against PEDV and can be tested in clinical studies for the development of veterinary drugs.

## Data Availability

All data generated or analyzed during this study are included in the manuscript.

## Abbreviations

PEDV: Porcine epidemic diarrhea virus
3CL protease: 3-chymotrypsin-like protease
MD: Molecular dynamics
RMSD: Root-mean-square deviation
RMSF: root-mean-square fluctuation
Rg: Radius of gyration
SASA: Solvent-accessible surface area
HBs: Hydrogen bonds
PCA: Principal component analysis
FEL: Free energy landscape
MM-PBSA: Molecular mechanics Poisson–Boltzmann surface area
